# Comparative transcriptomics reveals specific responding genes associated with atherosclerosis in rabbit and mouse models

**DOI:** 10.1371/journal.pone.0201618

**Published:** 2018-08-01

**Authors:** Leilei Wu, Qianlan Yao, Ping Lin, Yixue Li, Hong Li

**Affiliations:** 1 School of Life Sciences and Biotechnology, Shanghai Jiao Tong University, Shanghai, China; 2 CAS Key Laboratory of Computational Biology, CAS-MPG Partner Institute for Computational Biology, Shanghai Institute of Nutrition and Health, Shanghai Institutes for Biological Sciences, University of Chinese Academy of Sciences, Chinese Academy of Sciences, Shanghai, China; 3 Collaborative Innovation Center of Genetics and Development, Fudan University, Shanghai, China; Max Delbruck Centrum fur Molekulare Medizin Berlin Buch, GERMANY

## Abstract

Mouse and rabbit are frequently employed species for atherosclerosis research. With respect to modeling human atherosclerosis, it has been observed that variations in phenotype under commonly used atherogenic conditions are partial or no congruence between two species. However, genome-wide molecular variations are still lacking. To understand the differences between rabbit and mouse in developing atherosclerosis, here from aspect of orthologs, we compared the genome-wide expression profiles of two species under the same atherosclerosis driven factors: high-fat diet or LDLR deficiency. Our results illuminated that: 1) LDLR-deficiency induced different gene expression changes in rabbit and mouse. WHHL rabbit had more significantly differential expressed genes and the most of genes were down-regulated. 2) Some genes and functions were commonly dysregulated in high-fat fed rabbit and mouse models, such as lipid metabolism and inflammation process. However, high-fat intake in rabbit produced more differentially expressed genes and more serious functional effects. 3) Specific differential expression genes were revealed for rabbit and mouse related with high-fat intake. In the aspect of lipoprotein metabolism, APOA4 and APOB was the major responding gene in rabbit and mice, respectively. The expression change of APOA4 and APOB in human atherosclerosis was more similar to rabbit, and therefore rabbit might be a better animal model for investigating human lipoprotein metabolism related diseases. In conclusion, our comparative transcriptome analysis revealed species-specific expression regulation that could partially explain the different phenotypes between rabbit and mouse, which was helpful for model selection to study atherosclerosis.

## Introduction

High cholesterol level, high blood pressure, smoking and inflammation are well-known risk factors for atherosclerosis [1–3]. Among them, lipid induced atherosclerosis is involved in multiple organs especially in liver. The circulating low-density lipoprotein (LDL) is absorbed by liver cells via low-density lipoprotein receptor (LDLR), and then the surplus LDL cholesterol is brought back by reverse cholesterol transport (RCT) [4] to liver for elimination and maintaining cholesterol balance. When LDLR is defective, as in human familial hypercholesterolemia homozygotes [5], altered metabolism of Very-low-density lipoprotein (VLDL) induced overproduction of LDL [6] and adequate LDLs accumulate in the susceptible arterial sites. In addition, the high-level cholesterol and its oxidation products play an essential role in the pathogenesis of atherosclerosis [7,8]. On the other hand, atherosclerosis is regarded as an inflammation disease [2,9]. Increased cholesterol intake makes liver switch from a resilient and adaptive organ to an inflammatory and pro-atherosclerotic form [2], which aggravates the process of atherosclerosis.

In order to fully understand the pathophysiology of atherosclerosis, mouse and rabbit models are commonly used to mimic human liver response and uncover the underlying molecular mechanisms [10,11]. Mouse is the most widely used animal model due to the advantages of well-known genetic background, easy breeding, low cost of maintenance and high genetic manipulation. However, some important discoveries in atherosclerosis are found by rabbit models instead of by mouse models. For example, the anti-atherosclerotic effect of statins is verified in Watanabe heritable hyperlipidemic (WHHL) rabbits, but ineffective in mice [12]. Additionally, rabbit is more sensitive to high cholesterol [13]. LDLR-deficiency or 4-week high-fat diet can induce the atherosclerosis lesions in rabbit, which makes rabbit easily to be a model of atherosclerosis [14]. However, only simultaneous LDLR-deficient and high-fat diet in mouse can induce atherosclerosis in a short period [2,10,11]. Due to the pros and cons of both animal models, it is essential to understand the different molecular mechanism and decide the suitable research model.

Comparative transcriptome is an efficient method to reveal conserved and different aspects among species. The cores of comparative transcriptome analyses are ortholog genes, which evolved from a common ancestral gene via a speciation event [15] and retain the similar functions in the process of evolution [16]. Multiple approaches have been developed to compare the expression pattern of orthologs, such as differential expression analysis for finding differentially expressed genes (DEGs) [17], weighted gene co-expression network analysis (WGCNA) for identifying conserved sets of co-regulated genes [18], etc. Comparative transcriptome analysis could help to understand how novel phenotypes arise and maintain [18,19]. It therefore has been widely used in evaluating and comparing animal models [19] [20].

In our previous work, we had sequenced the genome and transcriptome of high-fat induced or LDLR-deficiency induced atherosclerotic rabbits [11], which makes the direct comparison of rabbit and mouse feasible. Here we explore the difference of rabbits and mice in generating atherosclerosis by comparative transcriptome analysis. Our results may help other researchers to select ideal animal models of atherosclerosis.

## Method

### Comparative analysis of normal liver from different species

The detailed workflow was shown in S1 Fig. Briefly, we search orthologs set in normal liver RNA-seq data among three species (human-rabbit-mouse). Then, we extracted intersection between orthologs set and atherosclerosis models expressing data for further analysis.

RNA-seq data of normal human livers (5 samples, ERR315327, ERR315394, ERR315414, ERR315451, ERR315463) and normal mouse livers (6 samples, ERR032203~ ERR032208) were downloaded from Expression Atlas. RNAs of normal rabbit livers (8 samples) were sequenced in our previous work [11]. All RNA-seq data were aligned to corresponding genome by tophat2 and gene expressions were calculated by cufflinks[21]. Genes with FPKM value larger than 0.5 were regarded as expressed in liver. The human/rabbit and human/mouse ortholog tables were downloaded from Ensemble database (v87). All rabbit transcriptome sequencing data has been submitted to the NCBI Sequence Read Archive by our previous work [11] with accession number SRP053164.

### Expression profiles of atherosclerosis animal models

Different atherosclerotic animal models had been established from rabbit or mouse by high-fat diet or LDLR deficiency. RNA sequencing of rabbit livers was performed in our previous work [11], involving four samples in each of the four experimental groups: New Zealand White high-fat (NZW HF), NZW low-fat (NZW LF), Japanese White low-fat (JW LF), and WHHL low-fat (WHHL LF) [11]. WHHL rabbit is a LDLR deficient animal model for hypercholesterolemia and is created by Dr. Yoshio Watanabe[22]. The mouse liver expression array data were directly downloaded from GEO dataset (GSE363), involving three samples in each of the four experimental groups: LDLR+/+ low-fat (LDLR+/+ LF), LDLR+/+ high-fat (LDLR+/+ HF), LDLR−/− low-fat (LDLR−/− LF), and LDLR−/− high-fat (LDLR−/− HF)[10].

In order to remove the bias between different analysis methods, we used Limma package to analysis RNA-Seq and expression array. Limma package is originally designed for microarray data[23], which let the mouse liver expression data perfectly fit. For RNA-Seq data, the raw read counts were generated by HTSeq. To adjust for heteroscedasticity of raw counts, the mean-variance relationships were modeled by the voom function[24] to obtain appropriate weights (S2 Fig). These weighed expression values were used in the subsequent Limma analysis.

### Differential expression analysis

Samples were classified into case and control based on experimental conditions and disease states. To investigate the effects of diet, three groups were analyzed: “LDLR+/+ HF vs. LDLR+/+ LF in mouse”, “LDLR-/- HF vs. LDLR-/- LF in mouse”, and “NZW HF vs. NZW LF in rabbit”. To investigate the effects of LDLR dysfunction, two groups were analyzed: “LDLR-/- LF vs. LDLR+/+ LF in mouse” and “WHHL LF vs. JW LF in rabbit”. In mouse, “LDLR-/- LF” did not have significant lesion; both “LDLR+/+ HF” and “LDLR-/- HF” got significant atherosclerosis lesions after 12 weeks feeding, while the lesion burden of “LDLR-/- HF” was the largest one among all conditions in mouse[10]. In rabbit, both “NZW HF” and “LDLR-deficient WHHL LF” got severe lesions. By applying linear regression based method in Limma packages, differential expression genes (DEGs) were calculated for the case and control samples in each group. Genes with fold change>2 and *P*<0.05 were regarded as statistically significant. To compare two groups under different experimental conditions, we compared the systematic expression change and the list of DEGs between groups.

### Functional enrichment analysis

In order to understand the relation between gene expression and experimental conditions, expression pattern and differential expression genes were respectively analyzed. Gene sets of GO (c5.bp.v5.2.symbols) and KEGG (c2.cp.kegg.v5.2.symbols) were downloaded from Molecular Signatures Database (MSigDB)[25]. GSEA was applied to rank gene and detect the top changed biological function terms[26]. The top 20 items were inputted into Cytoscape[26] to create a network ("enrichment map"). DAVID[27] v6.8 was used for functional enrichment analysis of differentially expressed genes.

### Other statistical analysis

Distance between two samples was defined as '1—Pearson correlation coefficient (PCC)'. For samples from the same species, PCC was calculated using expression values of all genes. For groups between different species, PCC was calculated using the expression fold change of orthologous genes. Hierarchical clustering was applied to illustrate the similarity between samples.

Expression array of normal human livers (GSE28893[28]) and livers of human coronary artery disease (CAD) patients (GSE40231[29]) were download from GEO database. Expressions of APOB and APOA4 were normalized by Z-score and then calculated the ratio of APOB and APOA4. All statistical analyses were performed in R 3.3.0.

## Results

### Orthologs and sample clustering

**Fig 1:**
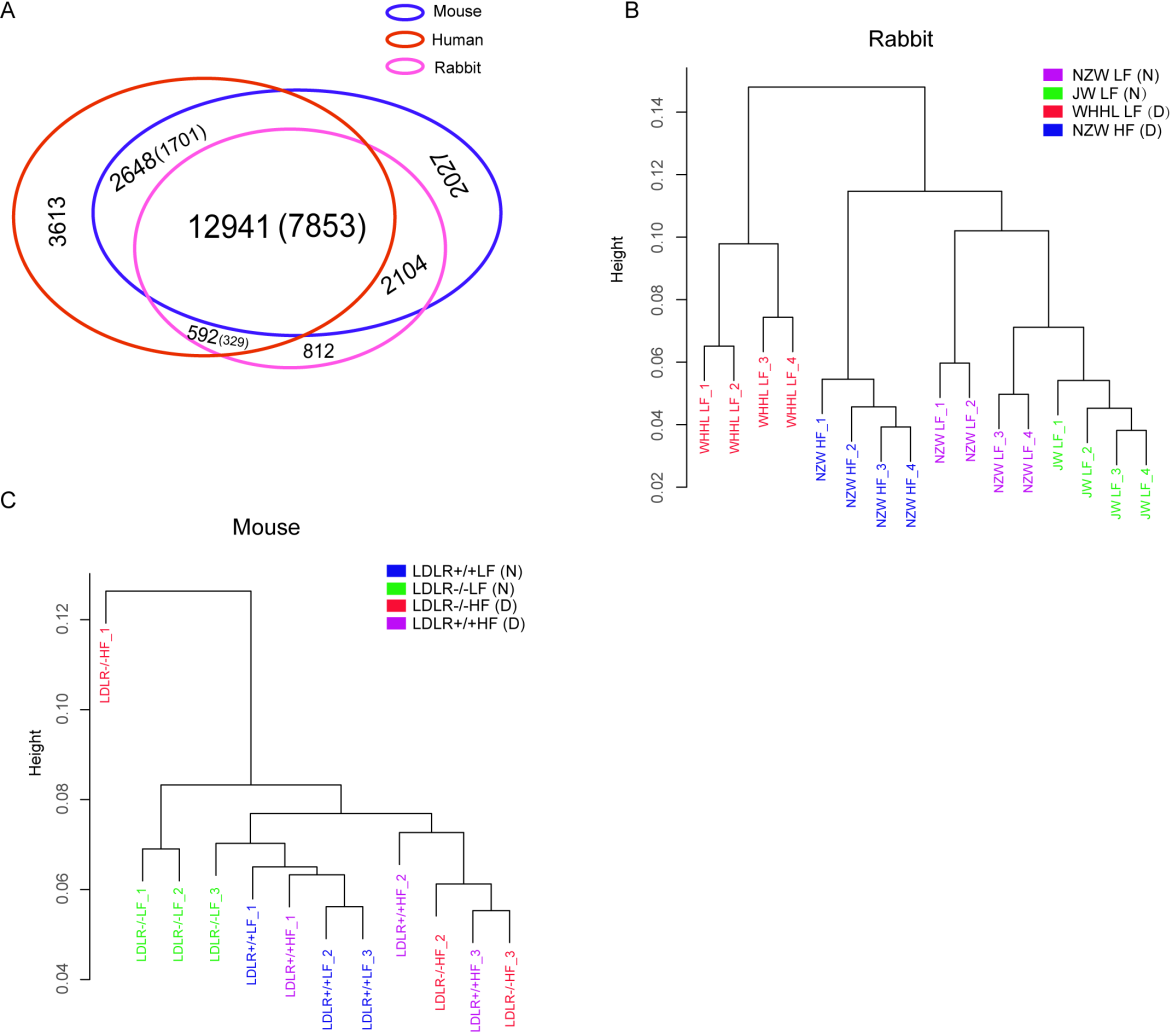
Overview of ortholog genes and liver expression profiles. (A) Comparison of the orthologs among human, mouse and rabbit. Numbers in parentheses are the number of orthologs that expressed in normal livers. (B) Cluster of rabbit liver samples based on all expressed genes in RNA-seq dataset. “D” and “N” in parentheses indicate samples came from atherosclerosis models or controls. (C) Cluster of mouse liver samples based on all expressed genes in microarray dataset.

We compared the genes among human, rabbit and mouse, and found 12941 orthologous genes. Among them, 7853 genes (60.68%) were commonly expressed in the normal livers of three species **(**Fig 1A). And 5938 of normal liver expressing orthologs were finally detected expression among cross-platform overlaps of atherosclerotic rabbit and mouse. Apart from commonly expressed orthologs among three species, we found 329 of 592 human-rabbit specific orthologs and 1701 of 2684 human-mouse specific orthologs expressed in normal livers. The number of human-mouse specific orthologs was larger than human-rabbit specific orthologs, which might be caused by the incomplete annotation of rabbit genome. Further, we investigated whether the previously reported human CAD related genes [11] were belong to human-rabbit specific orthologs or human-mouse specific orthologs. Only CETP was specific orthologs for human and rabbit but not for human and mouse. CETP encode cholesteryl ester transfer protein, which plays important roles in RCT and promotes the transfer of cholesteryl esters from antiatherogenic high-density lipoprotein (HDLs) to proatherogenic APOB–containing lipoproteins[11,30].

Next, sample distances were explored for rabbit and mouse separately. Firstly, based on all genes in each platform, we clustered the samples of each species based on Pearson correlation coefficients of all genes. Rabbit samples were clearly clustered into three groups, which was completely consistent with experimental conditions (Fig 1B). However, mouse samples clusters were not always following to experimental conditions (Fig 1C). Different clustering results indicated rabbit might be more sensitive to the experiment conditions than mouse. Secondly, 5938 orthologs expression profiles were used to cluster mouse and rabbit samples together. The result was consistent with the clustering of all genes (S3 Fig), which indicated the representative role of 5938 orthologs.

### Systemic expression change in different atherosclerotic models

**Fig 2:**
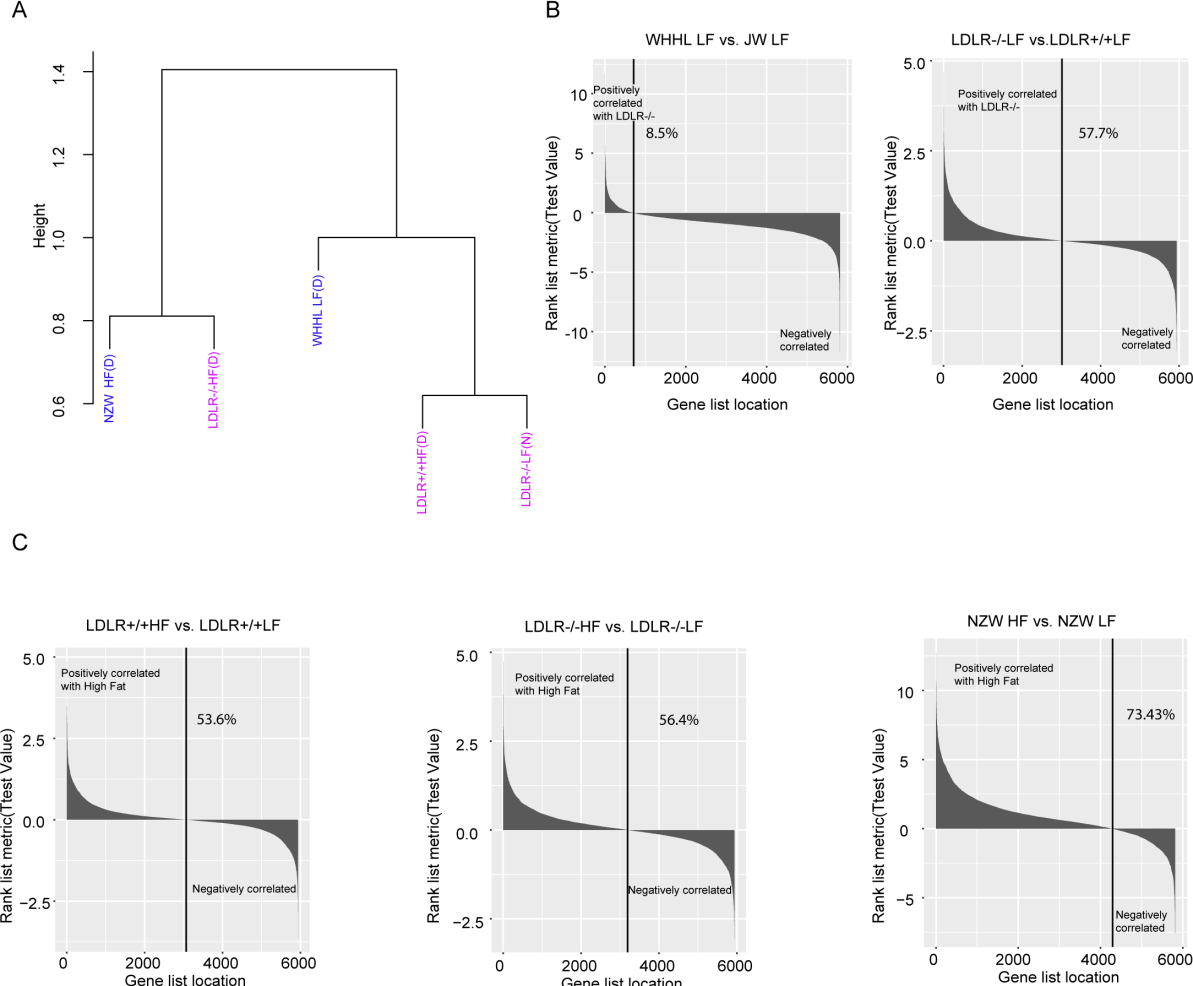
The global expression change in rabbit and mouse treatment groups. (A) Cluster of all treatment groups based on the expression fold change of orthologs. “LDLR+/+ HF” and “LDLR-/- HF” are the results of high-fat mouse compared to low-fat mouse; “NZW HF” is the result of high-fat rabbits compared to low-fat rabbits; “LDLR-/- HF” and “WHHL LF” are the results of LDLR-deficient mouse and rabbits compared to normal control. (B) The ranking metric which measures gene’s correlation with LDLR-deficiency induced phenotype. (C) The ranking metric which measures gene’s correlation with high-fat induced phenotype.

Previous studies show that high-fat diet and LDLR-deficiency induce different phenotypes in rabbits and mice [31,32]. To better understand the phenotypic difference, closeness of 5938 orthologs fold change expression profile and correlation of 5938 orthologs expression profile with conditions were explored. The similarities of three high-fat induced models (NZW HF, LDLR+/+ HF, LDLR-/- HF) and two LDLR-deficiency induced modes (WHHL LF, LDLR-/- LF) were illustrated in Fig 2A. When feeding with high fat, LDLR-/- mouse and NZW rabbits had a similar expression change. However, LDLR+/+ HF mouse were more similar with LDLR-/- LF mouse, far from LDLR-/- HF mouse. Besides, the expression change of WHHL LF was different from other four conditions. These results partly explained why high-fat diet or LDLR deficiency could not induce similar phenotype in mouse and rabbit.

In correlations aspect, nearly all genes were negatively (91.5%) related with LDLR deficiency in rabbits (Fig 2B left), while only 42.3% genes were negatively related with LDLR deficiency in mouse (Fig 2B right). For high-fat diet, the percentage of positively correlated genes gradually ascended in three experimental conditions: LDLR+/+ HF (53.6%), LDLR-/- HF (56.4%) and NZW HF (73.43%) (Fig 2C).

Then we compared the altered biological functions of different models. The three high-fat induced models shared two function clusters: sterol related process and immune/inflammation related process (S4 Fig). Among them, cytokine-cytokine receptor interaction was particularly interesting because of its essential roles in immune/inflammation. We found cytokine related pathways were activated among all three high-fat models. The leading edge analysis showed that different cytokines were activated in mouse and rabbit. Besides, the number of activated cytokines in rabbit was higher than that in mouse model (S4 Fig). Further differential expression analysis revealed significantly expressed cytokines in each model (S1 Table). We found that *IL15* and *IL18* were specifically upregulated in NZW HF rabbits, while the expressions of *CCL5 and IL12A/B* were significantly increased in high-fat mice. These results may be helpful to choose suitable animal model based on the specific cytokines. Apart from above shared function clusters, each kind of model enriched in special function clusters. Taking NZW HF rabbit as an example, mitochondrial dysfunction, extracellular matrix and cytoskeleton were significantly changed (S4 Fig). Almost all genes in electron transport chain were down-regulated in NZW HF rabbits (S4 Fig).

### Comparison of differentially expressed genes among different atherosclerotic models

**Fig 3:**
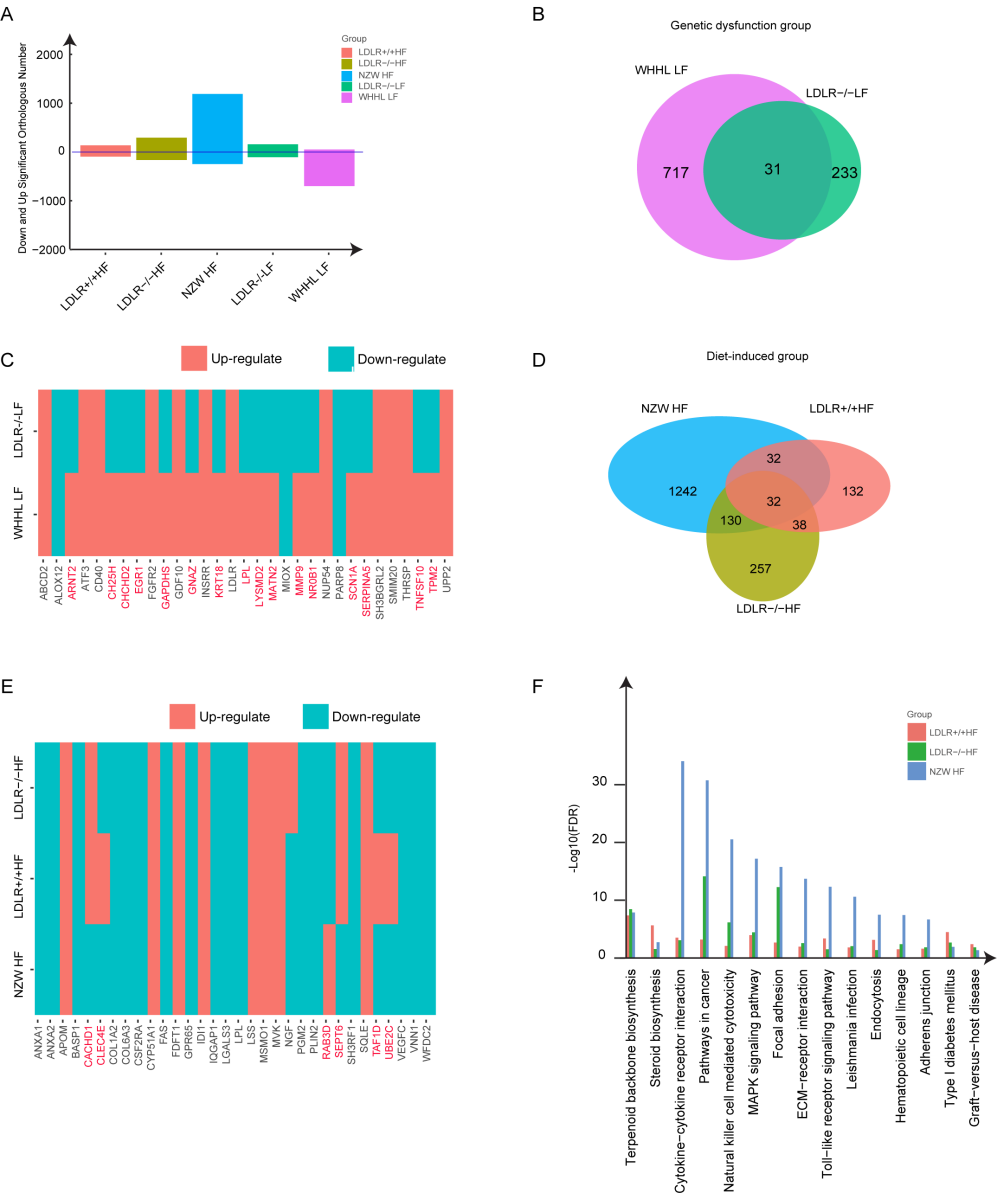
Comparison of differential expressed genes (DEGs) and enriched biological processes in rabbit and mouse models. (A) Number of up-regulated or down-regulated DEGs in different treatment groups. (B) Comparison of DEGs between two LDLR-deficient groups. (C) Changing directions of shared DEGs in LDLR-deficient rabbit and mouse. (D) Comparison of DEGs among three high-fat induced groups. (E) Changing directions of shared DEGs in three high-fat induced groups. (F) Shared biological processes enriched in three high-fat induced conditions in high-fat group.

In order to uncover the underlying mechanisms between rabbit and mouse responding to high-fat diet and LDLR-deficiency, differentially expressed genes (DEGs) were analyzed for each experiment condition. The numbers of DEGs were 234, 457, 1436, 264 and 748 for LDLR+/+ HF, LDLR-/- HF, NZW HF, LDLR-/- LF and WHHL LF samples, respectively. NZW HF rabbit had more DEGs than other conditions. In addition to the difference in the quantity, ratios of up-regulated gene number and down-regulated gene number were also different (Fig 3A). Mouse always displayed a half-to-half pattern for up- and down- regulated DEGs, while most DEGs were up-regulated in NZW HF rabbit and nearly all DEGs were down-regulated in WHHL LF rabbit. This trend was consistent with the results of all orthologs (Fig 2B and 2C).

Then we explored shared DEGs of rabbit and mouse under the same treatment. For LDLR-deficiency groups, 31 common DEGs were found between rabbit and mouse models (Fig 3B). However, 16 of them had opposite changing directions (red-labeled genes in Fig 3C). When we compared the altered functions of LDLR-deficiency rabbit and mouse, no function items were overlapped. This result might be related with the differences of hypercholesterolemia between LDLR-/- mouse and WHHL rabbit [10,11]. For high-fat diet induced groups, we found 32 shared DEGs in three models (Fig 3D) and 25 of them had the same changing direction (Fig 3E). These shared DEGs in the three high-fat diet induced models were further significantly enriched in lipid metabolism (cholesterol biosynthetic process, steroid biosynthesis), inflammation and immune related items, which indicated the core and common effects of high-fat intake to liver (Fig 3F).

### Species-specific expression regulation in lipid metabolism

**Fig 4:**
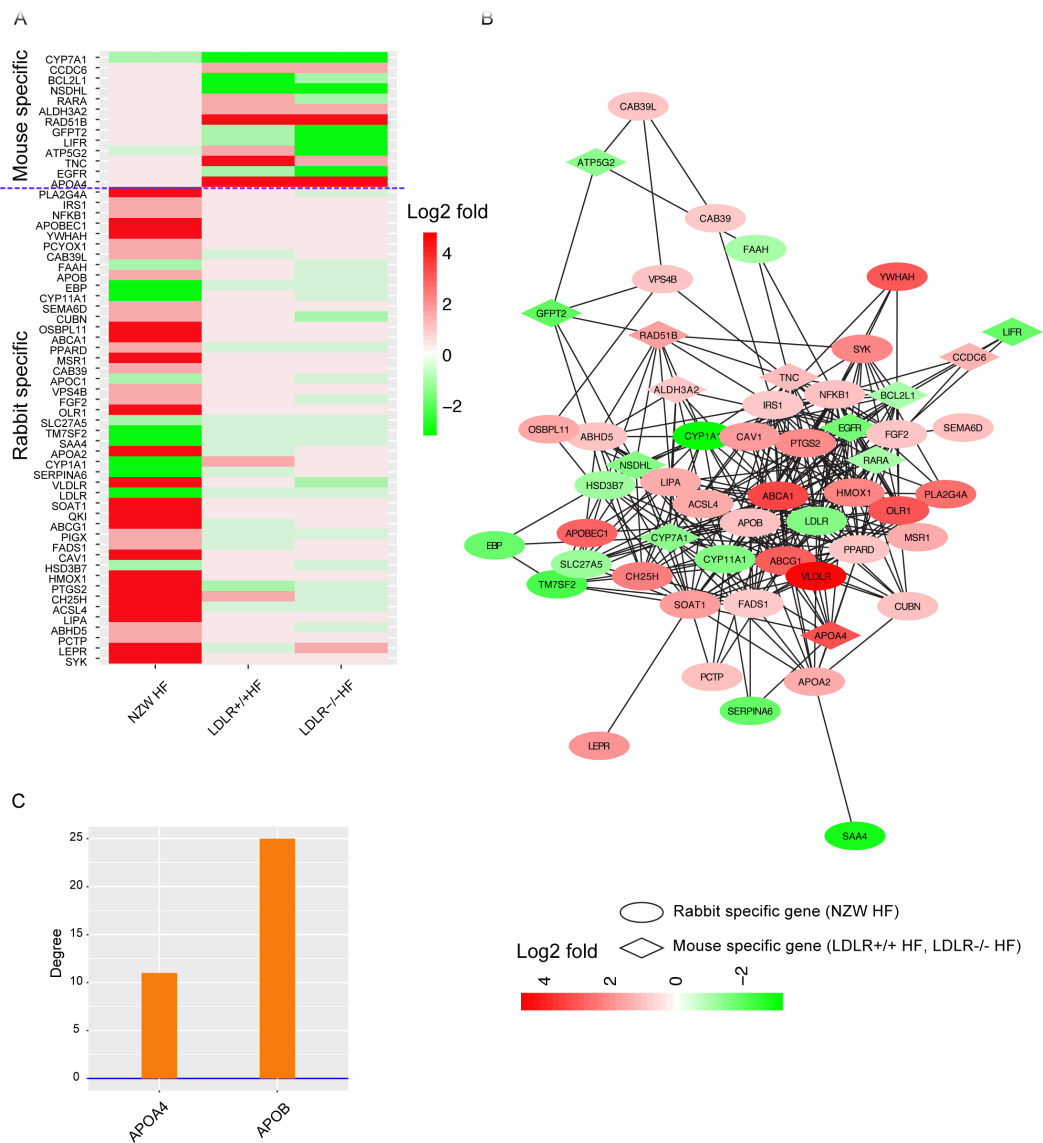
Species-specific genes in lipid metabolism. (A) Heatmap of mouse and rabbit specific DEGs in lipid metabolism. Red and green indicate log2 fold change value of up- and down- regulated genes, respectively. (B) Gene-gene interaction network of mouse and rabbit specific DEGs. Rabbit specific genes in ellipse and mouse specific genes in rhombus. All filled with red or green, which represented log2 fold change value of up- and down- regulated genes respectively. (C) Degrees of APOB and APOA4 in human protein-protein interaction network.

Due to the commonly dysregulated in lipid metabolism, we investigated molecular differences of mouse (LDLR+/+ HF, LDLR-/- HF) and rabbit (NZW HF) models in lipid metabolism. Totally 46 rabbit specific DEGs and 13 mouse specific DEGs and were found related with lipid metabolism (Fig 4A). Among rabbit specific DEGs, We observed that ABCA1, ABCG1, APOB were up-regulated and LDLR was down-regulated in rabbit, which indicated that rabbit liver might discharge cholesterol into blood and increase the total cholesterol level in blood[33]. Another Interesting gene is APOBEC1, which is apolipoprotein B (apoB) mRNA-editing enzyme and has been linked with cholesterol control (Fig 4A). Previous research showed that APOBEC1 is expressed in mouse liver[34], but extremely lowly expressed in human and rabbit liver[35,36]. Here our data showed that APOBEC1 was lowly expressed in the liver of NZW LF, JW LF and WHHL LF rabbits (FPKM<2), but upregulated in the liver of NZW HF rabbit (FPKM = 8, S4 Fig). High-fat induced upregulation of APOBEC1 and its roles in rabbit atherosclerosis is worth further study. Among mouse specific DEGs, APOA4 was the top one gene significantly up-regulated in two high-fat induced mouse models, which in turn reduces hepatic lipid burden by expanding VLDL [37]. The down regulation of NSDHL suggested cholesterol synthesis was limited. And High expression of ALDH3A2 that involves in the oxidation of medium- and long-chain aliphatic and aromatic aldehydes [38] might relief the oxidation damage in mouse liver. High expression of ALDH3A2 might relief the oxidation damage in mouse.

In order to better understand the relationship among species-specific DEGs, we mapped both mouse-specific and rabbit-specific DEGs to human protein-protein interaction network based on STRING database (Fig 4B). We calculated the degree of each gene, which is the number of connections that this gene interacts with others. We noted that the degree of APOB was 2.08 times higher than that of APOA4 (Fig 4C), indicating the core roles of APOB in human lipid metabolism. APOB and APOA4 were upregulated in atherosclerotic rabbit and mouse models respectively. Therefore, lipid metabolism of rabbit was more disordered by disrupting more gene interactions (Fig 4B).

**Fig 5:**
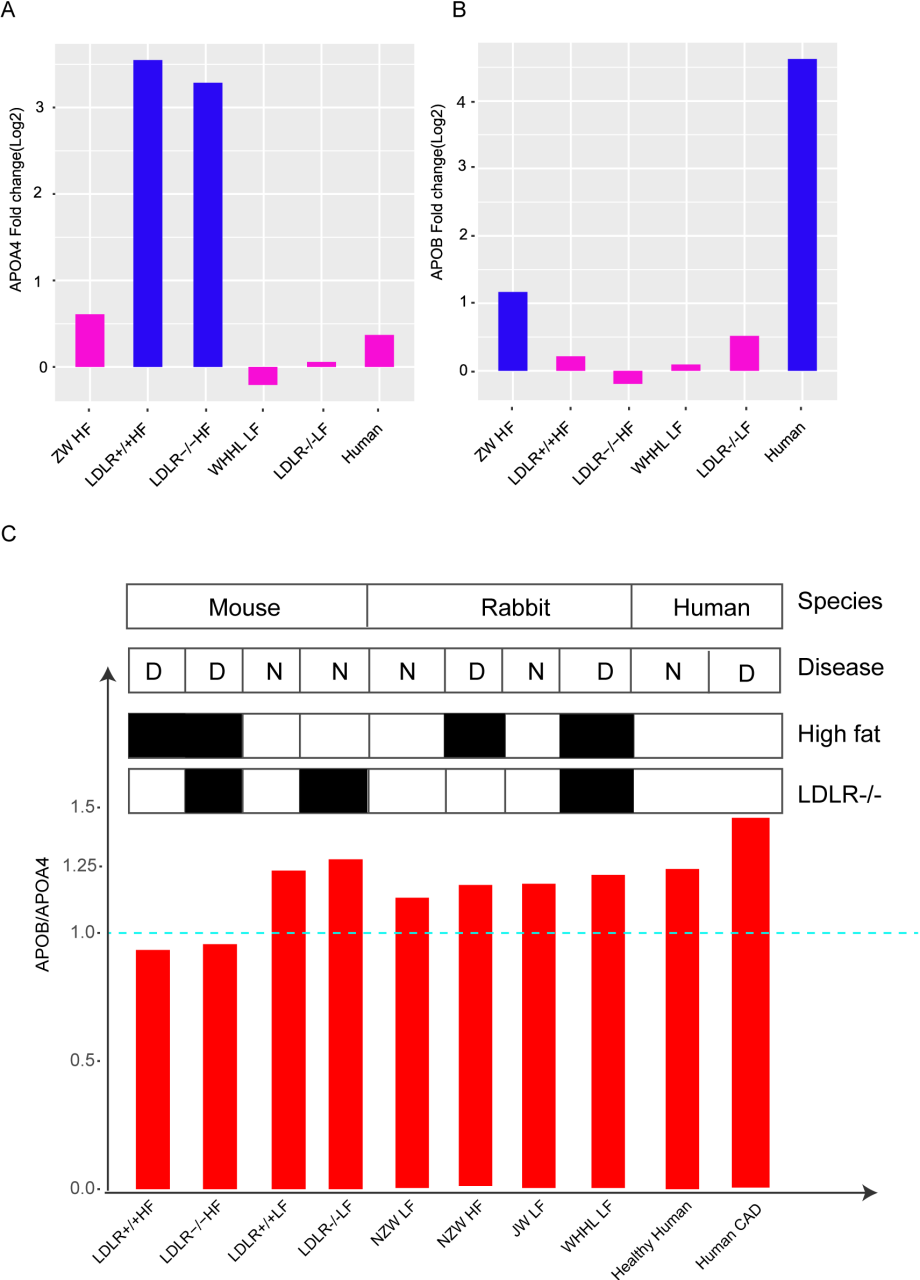
Expression pattern of APOB and APOA4 in rabbit, mouse and human. (A) APOA4 fold change in atherosclerotic animal models and human atherosclerotic patients. Blue bars indicate expression increases more than two times in disease groups. (B) APOB fold change in atherosclerotic animal models and human atherosclerotic patients. (C) Expression ratio between APOB and APOA4 in all available samples.

Furthermore, we compared the expression change of APOB and APOA4 among animal models and human CAD patients. APOA4 was specifically up-regulated in the liver of LDLR+/+ HF and LDLR-/- HF mouse, while its expression did not significantly change in human patients and rabbit models (Fig 5A). APOB significantly increased in human atherosclerotic patients. Only high-fat fed NZW rabbits had up-regulated APOB while other models did not (Fig 5B). To further compare the expression status of APOB and APOA4 in every single model, their expression ratios (APOB/APOA4) were calculated (Fig 5C). Result showed that APOB/APOA4 was higher than 1 in LDLR+/+ LF and LDLR-/- LF mouse, while high fat intake made this ratio down to 1. However, the ratio of APOB/APOA4 was higher than 1 no matter in normal rabbits, atherosclerotic rabbits, healthy human, or atherosclerotic patients. This result indicated that high-fat fed rabbit model better mimic the human atherosclerotic patients in cholesterol and lipoprotein metabolism.

## Discussion

To our knowledge, this is the first study to directly compare the transcriptome of rabbit and mouse atherosclerotic models. We found different expression patterns between two animal models and revealed specific DEGs related to lipid metabolism for both animal models. Additionally, compared to mouse, the primary responding molecules *(*APOA4 and APOB) had more similar expression change in high-fat fed rabbits and human atherosclerotic patients, suggesting rabbit is a better model to simulate the lipoprotein metabolism of human atherosclerosis.

Previous research shows that *APOB* has two isoforms (apoB-48 and apoB-100) created by APOBEC1 in mouse liver[34], while previous studies find no apoB-48 is formed in the liver of human and rabbit. APOBEC1 is believed to express only in small intestine in human and rabbit[39], which is consistent with low or not any expression value in all other tissues in rabbit here. An interesting finding was that after high fat intake in NZW rabbit a significant increase of APOBEC1 expression. This phenomenon might be explained by ectopic expression induced by high fat intake. In human, previous researches show that ectopic expression of APOBEC1 occurs in hepatocellular carcinoma[40], lung carcinoma[41] etc. This ectopic expression is not necessary functional[40,42]. Here in rabbit, whether APOBEC1 plays its role and the underlying regulation mechanism remain further study. Besides, whether this kind of expression also happens in human under same factor remained detailed study. Different from apoB-100, apoB-48 in mice makes VLDL not converted to LDLs and metabolized like chylomicron remnants, thus the double knockout of LDLR and APOE can make mouse get severe hypercholesterolemia[43]. However, absence of apoB-48 makes rabbit more sensitive to high fat intake or LDLR deficiency. When LDLR defects in WHHL rabbit, nearly all genes in liver were suppressed and severe atherosclerosis occurred in aorta. LDLR was also significantly down-regulated after high fat fed, which showed that rabbit had no efficient backup transportation system like mouse.

For another aspect, HDL is predominant lipoprotein in mouse while VLDL, LDL in rabbit [13]. And in rabbit, a high cholesterol diet induces the production of large cholesterol-ester rich beta-VLDL (intermediate between VLDL and LDL), which becomes the predominant lipoprotein and contributes to foam cell formation in macrophages. HDL is inversely related with atherosclerosis and helpful in RCT. RCT promotes the efflux of excess cholesterol from peripheral tissues and returns it to the liver for biliary excretion[4], which prevent cholesterol, LDL oxidation[44] and relive atherosclerosis. Additionally, mouse genome lacks CETP, which is an important enzyme in human and rabbit RCT and transfers cholesteryl esters from antiatherogenic HDLs to proatherogenic APOB–containing lipoproteins[30]. Therefore, rabbit may accumulate higher LDL and VLDL cholesterol than mouse after high-fat fed.

We also noted that there existed some limitations in rabbit and mouse datasets. The experiment conditions of two animal models were not completely consistent, and the transcriptome platforms were different. To eliminate the effects of this difference, we focused on the expression change between atherosclerosis and control groups, instead of the absolute expression value. In future, we plan to establish atherosclerosis rabbit and mouse models, and measure dynamic expression profiles using RNA-Seq. Through time series analysis between two animal models, we expect to verify key molecules that determine the formation of atherosclerotic lesion.

Our results showed common and different expression profiles between atherosclerosis rabbit and mouse models, which are helpful for model selection to study atherosclerosis. Additionally, the expression pattern of APOB and APOA4 under high-fat diet or LDLR-deficient conditions demonstrated that rabbit model is more similar with human in lipid metabolism and atherosclerosis.

## Supporting information

S1 Fig The workflow of this study. (TIF)

S2 Fig Relation between standard deviation(STD) and mean. (A) Mean and STD relation before linear modeling in rabbit; (B) Mean and STD relation after linear modeling in rabbit; (C, D) Mean and STD relation before and after linear modeling in mouse.
(TIF)

S3 Fig Cluster of all rabbit liver samples and mouse liver samples based on 5938 orthologs. (TIF)

S4 Fig Common and difference of biological functions in high-fat induced models and LDLR-deficient models between rabbit and mouse. (A) Function clusters based on top 20 biological functions in five conditions. Red dot represents positive relation with its own condition induced phenotype and blue represents negative relation with its own condition induced phenotype. (B) One shared KEGG pathway: cytokine cytokine receptor interaction in three conditions under high-fat groups. Top plot represents enrichment plot and bottom heatmap represents corresponding cytokines contributing to activate this pathway. (C) Clusters of cellular component functions located in of mitochondria in rabbit. (D) Fold change of genes in electron transportation chain among three conditions in high fat induced group. (E) FPKM value of APOBEC1 in all available tissues with different experiment conditions in rabbit.
(TIF)

S1 Table Significantly changed cytokines, chemokines and corresponding receptors. (XLSX)
