# Supplementary figures and images for: Comparative transcriptomics reveals specific responding genes associated with atherosclerosis in rabbit and mouse models
Source: PLoS One. 2018 Aug 1;13(8):e0201618. doi: 10.1371/journal.pone.0201618 (PMC6070260; doi:10.1371/journal.pone.0201618)

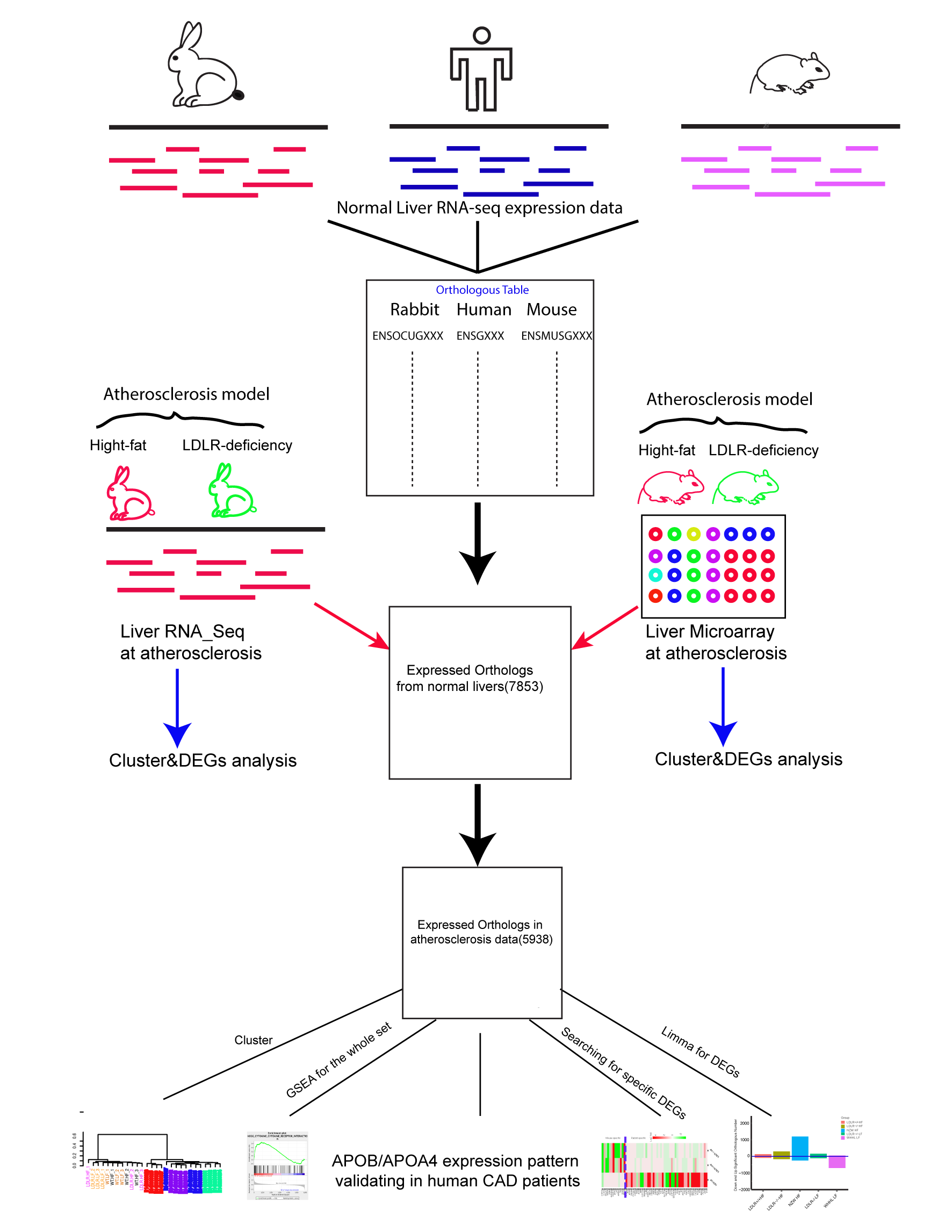

Supplement: S1 Fig — (TIF) [file pone.0201618.s001.tif]

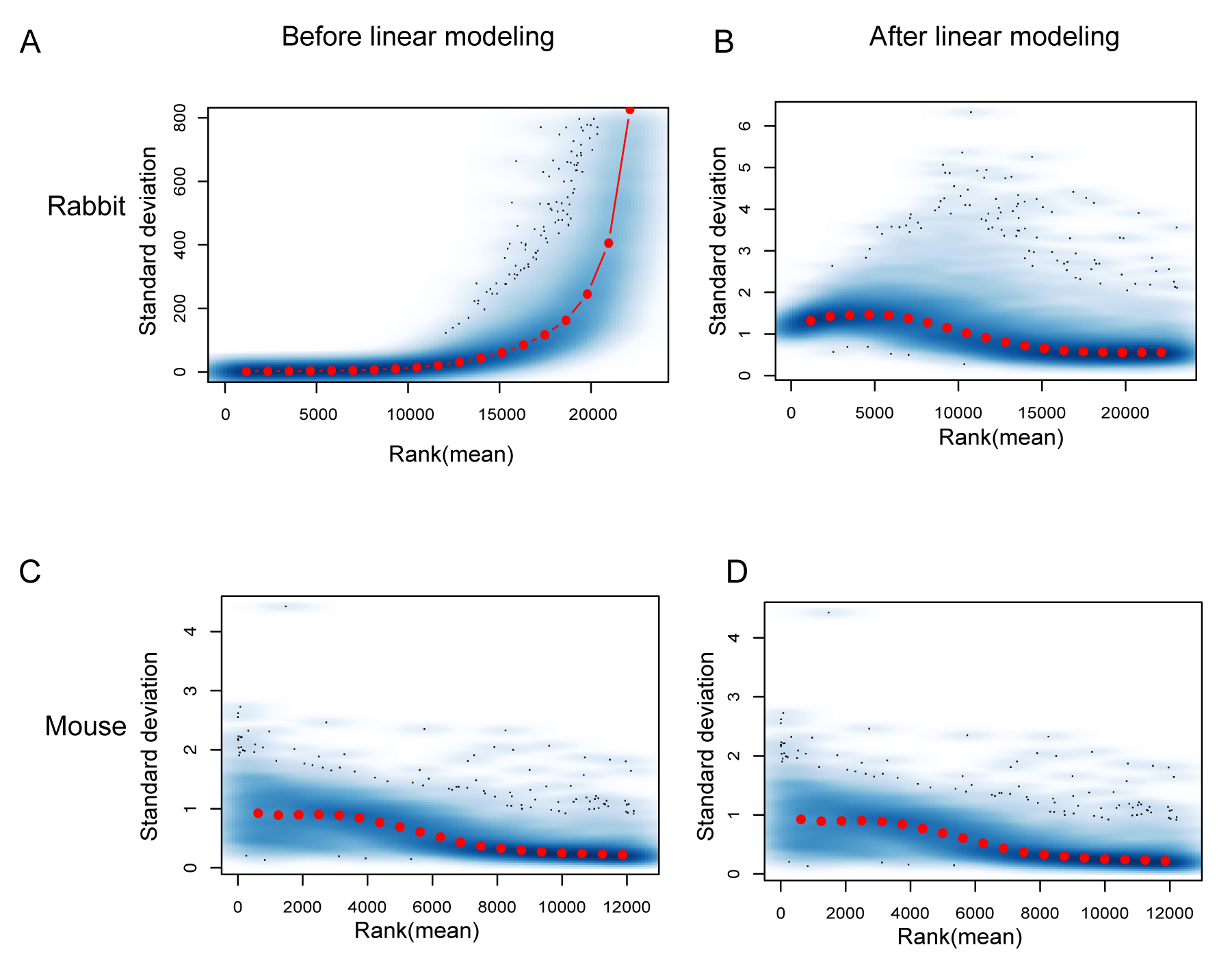

Supplement: S2 Fig — (A) Mean and STD relation before linear modeling in rabbit; (B) Mean and STD relation after linear modeling in rabbit; (C, D) Mean and STD relation before and after linear modeling in mouse. (TIF) [file pone.0201618.s002.tif]

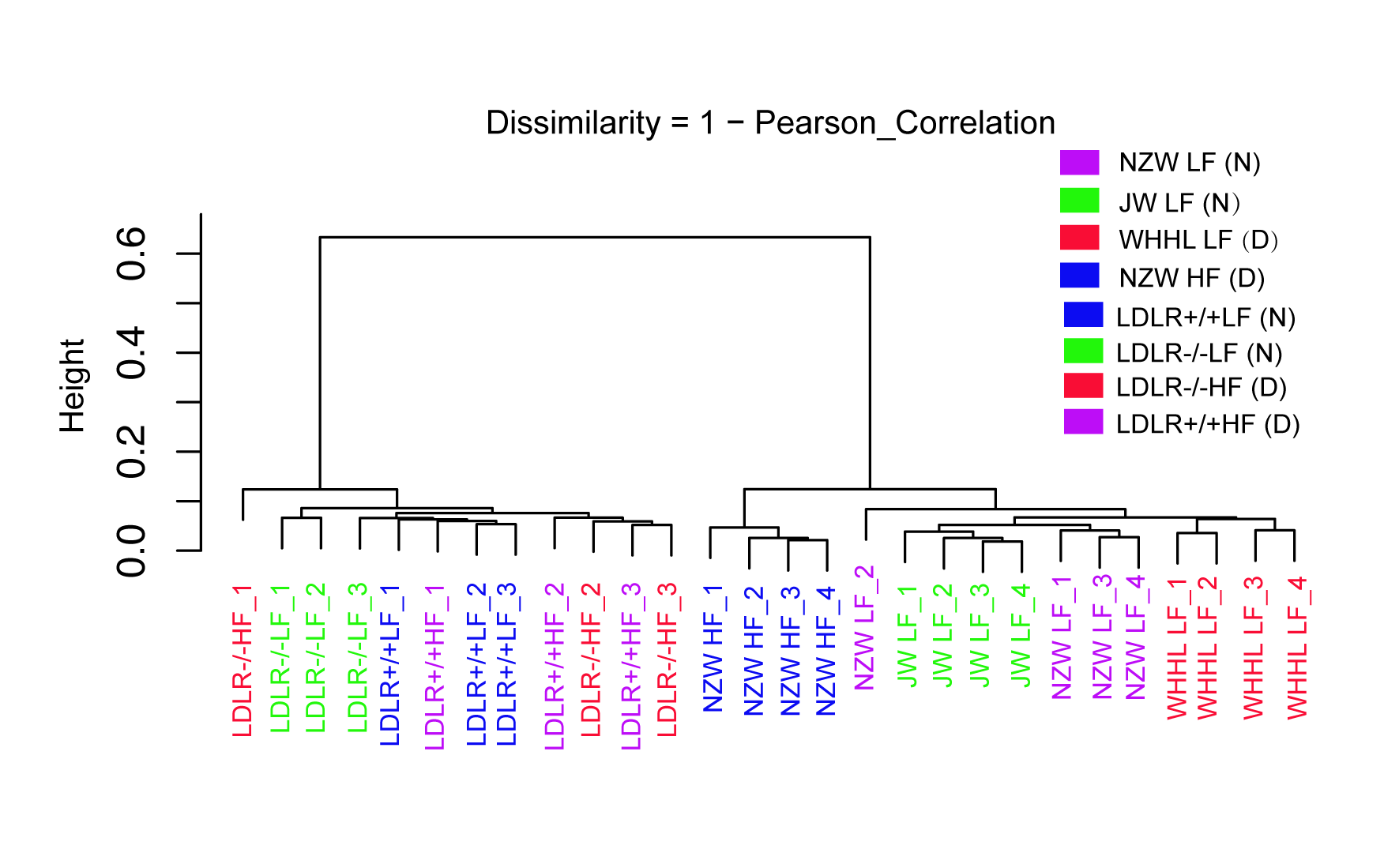

Supplement: S3 Fig — (TIF) [file pone.0201618.s003.tif]

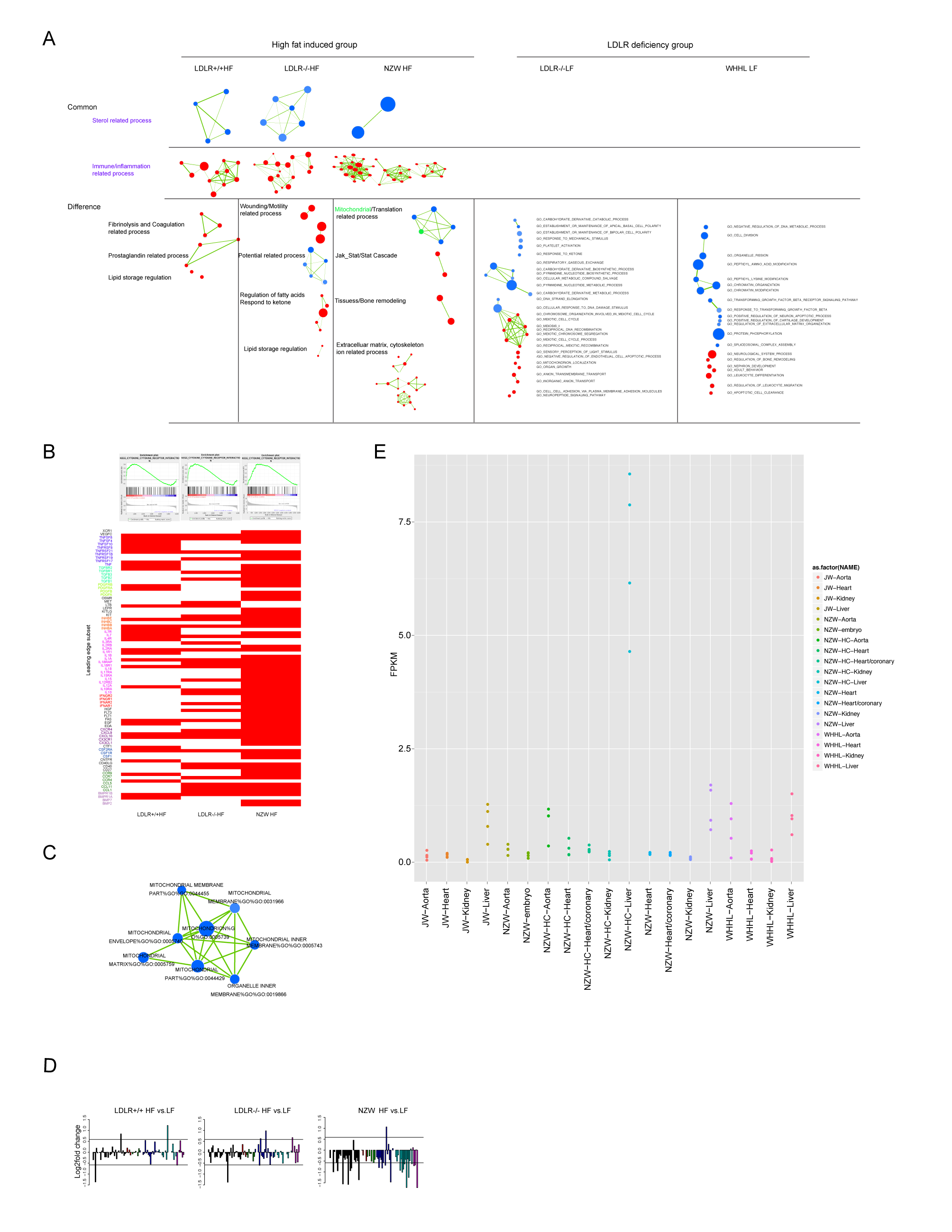

Supplement: S4 Fig — (A) Function clusters based on top 20 biological functions in five conditions. Red dot represents positive relation with its own condition induced phenotype and blue represents negative relation with its own condition induced phenotype. (B) One shared KEGG pathway: cytokine cytokine receptor interaction in three conditions under high-fat groups. Top plot represents enrichment plot and bottom heatmap represents corresponding cytokines contributing to activate this pathway. (C) Clusters of cellular component functions located in of mitochondria in rabbit. (D) Fold change of genes in electron transportation chain among three conditions in high fat induced group. (E) FPKM value of APOBEC1 in all available tissues with different experiment conditions in rabbit. (TIF) [file pone.0201618.s004.tif]
